# Integrated single-cell and bulk transcriptomic analyses reveal UTF1-associated spermatogenic dysfunction and identify ACTRT2 and AKAP4 as biomarkers in non-obstructive azoospermia

**DOI:** 10.3389/fgene.2026.1975399

**Published:** 2026-10-26

**Authors:** Jin Yu, Mingming Li, Yue Zhou, Hekun Liu, Chengbin Pei, Lianghong Ma

**Affiliations:** 1 The First Clinical Medical College, Ningxia Medical University, Yinchuan, China; 2 Department of Urology, General Hospital of Ningxia Medical University, Yinchuan, China; 3 Ningxia Human Sperm Bank, General Hospital of Ningxia Medical University, Yinchuan, China; 4 Institute of Medical Sciences, General Hospital of Ningxia Medical University, Yinchuan, China; 5 Key Laboratory of Fertility Preservation and Maintenance of Ministry of Education, Ningxia Medical University, Yinchuan, China

**Keywords:** biomarker, cell-cell communication, immune infiltration, non-obstructive azoospermia, single-cell RNA sequencing, spermatogenesis, UTF1

## Abstract

**Introduction:**

Non-obstructive azoospermia (NOA) is a severe male infertility disorder caused by spermatogenic failure, and UTF1 is critical for maintaining undifferentiated spermatogonia and regulating germ cell development. This study aimed to explore the role of UTF1-related spermatogenic dysfunction in NOA and identify potential diagnostic biomarkers.

**Methods:**

We integrated NOA transcriptomic datasets with single-cell RNA sequencing (scRNA-seq) data from Utf1 conditional knockout and normal mouse testes. Bioinformatic analyses and machine learning were applied to screen biomarkers, while functional enrichment, immune infiltration, cell-cell communication, and pseudotime trajectory analyses were performed together with histological validation in Utf1-deficient testes.

**Results:**

Spermatogonia, Sertoli cells, and spermatids were identified as key cell populations. ACTRT2 and AKAP4 were confirmed as consistently downregulated NOA biomarkers and were integrated into a well-calibrated nomogram. These two genes were associated with cytoskeletal remodeling and meiotic regulation. NOA was also characterized by altered immune infiltration and cell-cell communication, whereas Utf1 deficiency impaired testicular development and spermatogenesis.

**Discussion:**

Collectively, UTF1 deficiency was closely associated with spermatogenic dysfunction and microenvironmental remodeling in NOA, while ACTRT2 and AKAP4 might serve as potential diagnostic biomarkers of this process. These findings provide new insights into the molecular basis of NOA and may support future disease stratification and targeted intervention strategies.

## 1 Introduction

Male infertility is a significant global public health issue. Approximately 7% of men worldwide suffer from infertility (Tang et al., 2022), with azoospermia representing one of the most severe clinical phenotypes (Eisenberg et al., 2023; Mazzilli et al., 2023). Among azoospermia cases, non-obstructive azoospermia (NOA) accounts for up to 70% (Wang et al., 2025e), and the etiology remains unknown in the majority of patients (Fang et al., 2024; Wang J. et al., 2025). Currently, although testicular sperm extraction combined with intracytoplasmic sperm injection (TESE-ICSI) enables some patients to achieve fertility (Yao et al., 2025), there is still no effective treatment for patients who completely lack mature spermatozoa within the testis. Therefore, a systematic elucidation of the molecular and cellular mechanisms underlying NOA is of great significance for deepening our understanding of the pathogenesis of NOA and developing novel diagnostic and therapeutic strategies.

Spermatogenesis is a complex process in which spermatogonial stem cells differentiate into mature spermatozoa, relying on a finely regulated microenvironment orchestrated by interactions between germ cells and supporting cells, such as Sertoli cells (Rajachandran et al., 2023; Liu W. et al., 2024). This process encompasses multiple stages, including cell proliferation, differentiation, meiosis, and spermiogenesis, all of which are tightly controlled by transcriptional regulation and coordinated intercellular signaling networks (Han, 2024; Ishiguro, 2024; Kaltsas et al., 2026). Studies have shown that NOA is not only associated with defects in germ cell differentiation but also closely related to dysfunction of supporting cells, alterations in the immune microenvironment, and imbalances in intercellular communication (Abroudi et al., 2025; Jiang et al., 2025; Wang S. et al., 2025). Given the involvement of multiple functionally distinct cell populations, the high degree of heterogeneity among different cell types involved in spermatogenesis further complicates the elucidation of disease mechanisms, underscoring the need for high-resolution approaches capable of resolving cellular diversity at the single-cell level (Tirumalasetty et al., 2024; Cui et al., 2025).

UTF1 is a transcriptional regulator closely associated with the maintenance of pluripotency. It is highly expressed in pluripotent stem cells and exhibits a specific expression pattern during early embryonic development and in primordial germ cells, where it is thought to participate in maintaining stem cell states and regulating cell fate transitions (Raina et al., 2025). In testicular tissue, UTF1 is primarily localized in undifferentiated spermatogonia and is considered one of the key marker molecules for early undifferentiated spermatogonia/spermatogonial stem cells in humans (Ba Omar et al., 2025). Recent studies suggest that UTF1 is closely involved in the developmental program of germ cells, and its deficiency or dysregulated expression may affect the maintenance of spermatogonia, the transition of differentiation states, and subsequent spermatogenesis (Kasowitz et al., 2017; Zhu et al., 2024). UTF1 not only participates in regulating stem cell self-renewal and proliferation but also cooperates with core pluripotency factors such as Oct4 and Sox2 to maintain the differentiation potential of germ cells (Laskowski and Knoepfler, 2012; Roshan et al., 2024). In patients with NOA, UTF1 expression is dysregulated and accompanied by altered spatial localization, suggesting that it may play an important regulatory role in disease pathogenesis (Roshan et al., 2024). However, since global knockout of *Utf1* results in embryonic lethality (Kasowitz et al., 2017), tissue-specific conditional knockout models are essential for investigating its *in vivo* functions; to date, such models remain lacking, and the specific molecular mechanisms by which UTF1 regulates spermatogenesis and its precise role in NOA pathogenesis remain to be elucidated.

To address these gaps, the present study generated a testis-specific conditional knockout mouse model of *Utf1* and conducted systematic analyses ranging from *in vivo* phenotypic characterization to single-cell transcriptomic profiling, thereby comprehensively investigating the effects of *Utf1* deficiency on testicular development and spermatogenic function. Furthermore, by integrating mouse single-cell data with human NOA transcriptomic data, we screened and validated potential key molecular biomarkers, aiming to elucidate the UTF1-dependent mechanisms underlying spermatogenic impairment and to provide a theoretical framework for the precise diagnosis and targeted intervention of NOA.

## 2 Materials and methods

### 2.1 Data acquisition

Transcriptomic data were retrieved from the Gene Expression Omnibus (GEO) public repository. The training cohort was built upon the GSE145467 dataset (platform: GPL4133), encompassing testicular transcriptomic profiles from 10 NOA patients and 10 fertile controls. An independent validation cohort was derived from the GSE45887 dataset (platform: GPL6244), comprising 16 NOA and 4 control testicular tissue samples. Complementarily, scRNA-seq data were produced through in-house experiments on mouse testicular tissues, covering 3 wild-type controls (Ctrl) and 3 *Utf1*-knockout (*Utf1* ^cKO^) samples.

### 2.2 Processing of scRNA-seq data and cell annotation

The “Seurat” package (v 5.1.0) (Hao et al., 2024) was adopted for single-cell expression data processing. Quality filtering was applied according to the following thresholds: genes detected in three or fewer cells were discarded; cells with fewer than 200 or more than 6,000 detected genes (nFeature_RNA) were removed; cells in which mitochondrial transcripts exceeded 20% of total counts were excluded; cells with UMI counts (nCount_RNA) surpassing 20,000 were eliminated. Post-filtering expression matrices were normalized *via* the NormalizeData function. The FindVariableFeatures function with VST method was then applied to select the top 2,000 highly variable genes (HVGs) for downstream processing. PCA was conducted on this HVG set, with the optimal PC number determined by two complementary criteria: permutation-based statistical validation through the JackStraw test (P < 0.05) and the elbow point of the scree plot. Cell clustering was achieved through FindNeighbors and FindClusters functions applied to the retained PCs (resolution = 0.07). Cluster identities were assigned by cross-referencing marker gene signatures from the CellMarker 2.0 database and published literature (Wang Q. et al., 2025; Yang J. et al., 2025).

### 2.3 High-dimensional weighted gene co-expression network analysis (hdWGCNA)

Building on scRNA-seq annotation results, spermatogenesis-relevant cells were isolated, and the “hdWGCNA” package (v 0.4.03) (Morabito et al., 2023) was employed to construct co-expression networks and extract spermatogenesis-related genes. The Seurat object was initialized for WGCNA *via* the SetupForWGCNA function; metacells were aggregated using MetacellsByGroups, and the TestSoftPowers function was leveraged to determine the optimal soft-thresholding power (scale-free topology fit R^2^ ≥ 0.80; mean connectivity <200). Network construction was executed through the ConstructNetwork function. Following removal of the unassigned grey module, the top 10 genes per module, ranked by descending module membership (kME) scores, were extracted. Modules whose constituent genes were significantly concentrated in spermatogenesis-associated cell types were designated key modules. Per key module, the top 100 genes by kME were retained (Wang et al., 2025d) as key module-related genes (KMRGs). Human one-to-one orthologs of mouse KMRGs were mapped using the “biomaRt” package (v 2.56.1) (Durinck et al., 2009), yielding human spermatogenesis-related genes for all subsequent analyses.

### 2.4 Differential expression analysis and candidate gene screening

Differential gene expression between NOA and control specimens was assessed in the GSE145467 dataset using the “limma” package (v 3.56.2) (Ritchie et al., 2015), with the criteria: P. adj <0.05 and |log_2_FC| > 1. The resulting DEG list was intersected with human KMRGs using the “ggvenn” package (v 0.1.10) (Zheng et al., 2022), and genes residing at this intersection were designated candidate genes.

### 2.5 Functional enrichment analysis and protein-protein interaction (PPI) network analysis

Functional enrichment analysis was carried out to illuminate biological processes and pathways associated with candidate genes. GO and KEGG pathway analyses were performed through the “clusterProfiler” package (v 4.2.2) (Yu et al., 2012) at a significance threshold of P < 0.05. To resolve protein-level interaction relationships, candidate genes were submitted to the STRING database for PPI prediction (interaction score ≥0.15). Isolated nodes were pruned from the resulting network, and the refined PPI map was rendered in Cytoscape (v 3.9.1) (Shannon et al., 2003).

### 2.6 Identification of candidate biomarkers

To further screen the candidate genes, the MCODE plugin (https://apps.cytoscape.org/apps/mcode) was used to identify key subnetworks with default parameter settings: Degree Cutoff = 2, Node Density Cutoff = 0.1, Node Score Cutoff = 0.2, K-Core = 2, and Max. Depth = 100. Genes within the subnetwork with the highest score were defined as feature genes. Three machine learning algorithms were subsequently applied for feature selection. First, Support Vector Machine-Recursive Feature Elimination (SVM-RFE) was utilized to iteratively eliminate the least important features using the “e1071”package (v 1.7.16) (Shi et al., 2023) with 5-fold cross-validation. Next, a Random Forest (RF) classifier was implemented to evaluate feature importance based on the Mean Decrease Gini coefficient using the “randomForest” package (v 4.7.1.2) (Wang et al., 2023) (ntree = 500), retaining the top 10 genes with the highest scores. Furthermore, the Boruta algorithm was applied to assess gene importance using the “Boruta” package (v 8.0.0) (Yan et al., 2024) (pValue = 0.01, maxRuns = 100), and genes labeled “Confirmed” were preserved. Finally, intersection analysis of the genes screened by the three algorithms was performed using the “ggvenn” package (v 0.1.10), yielding candidate biomarkers. The models constructed in the training cohort were further applied to the independent validation cohort GSE45887 for external validation. Model performance was evaluated by area under curve (AUC), accuracy, Kappa coefficient, sensitivity, specificity, Positive predictive value (PPV), negative predictive value (NPV), and F1 score.

### 2.7 Biomarker screening and construction and evaluation of a nomogram

Expression differences of candidate biomarkers between NOA and control groups were tested in both GSE145467 and GSE45887 datasets using the Wilcoxon rank-sum test (P < 0.05). Candidates demonstrating statistically significant and directionally consistent differential expression across both cohorts were confirmed as biomarkers. In addition, UTF1 expression was compared between NOA and control samples in both the GSE145467 and GSE45887 datasets using the Wilcoxon rank-sum test (P < 0.05). A nomogram was subsequently developed using the “rms” package (v 6.8.1) (Lin et al., 2024) to quantify the predictive value of these biomarkers for NOA. Calibration accuracy was assessed through calibration curves generated *via* the “regplot” package (v 1.1.0) (Sui et al., 2022), and model fit was formally evaluated by the HL goodness-of-fit test, where P > 0.05 indicated adequate concordance between predicted probabilities and observed outcomes.

### 2.8 Genomic and subcellular localization and functional pathway analysis

Chromosomal mapping of the biomarkers was visualized using the “RCircos” package (v 1.2.2) (Zhang et al., 2013). Potential intracellular functional sites were inferred through subcellular localization predictions retrieved from the GeneCards database. GSEA was conducted on the GSE145467 cohort to characterize disease-associated pathways differentiating NOA from controls, with the reference gene set “c2. cp.kegg_medicus.v2025.1. Hs.symbols.gmt” sourced from MSigDB. Gene ranking was based on Spearman correlation coefficients between individual genes and each biomarker. GSEA was executed *via* the “clusterProfiler” package (v 4.2.2), applying thresholds of P. adj <0.05 and |NES| > 1.

### 2.9 Immune infiltration analysis

To characterize immune microenvironmental dynamics across NOA progression, the ssGSEA algorithm embedded in the “GSVA” package (v 1.53.28) (Hänzelmann et al., 2013) was applied to the GSE145467 dataset for quantifying the relative abundance of 28 immune cell types. Cell-type signatures were sourced from the TISIDB database and evaluated across both NOA and control samples. Between-group differences in infiltration levels were assessed by Wilcoxon rank-sum testing, with immune subsets reaching P < 0.05 classified as differentially infiltrated. Spearman correlation analysis, performed *via* the “psych” package (v 2.2.9) (Orifjon et al., 2023), was then used to map pairwise associations between biomarkers and these differentially infiltrated subsets (|cor| > 0.3, P < 0.05).

### 2.10 Construction of regulatory networks and drug prediction

Post-transcriptional regulatory interactions of the biomarkers were explored by predicting targeting miRNAs *via* the TarBase database. Upstream transcription factors (TFs) governing biomarker expression were queried from the hTFtarget database. An integrated miRNA-mRNA-TF regulatory network was subsequently assembled and rendered in Cytoscape (v 3.9.1). Drug-gene interaction pairs targeting the identified biomarkers were retrieved by querying the Drug Signatures Database (DsigDB) for potential therapeutic compound identification.

### 2.11 Identification of key cells and cell-cell communication analysis

To identify key cell types closely associated with the biomarkers, the cell counts and proportional distributions of biomarker-expressing cells were quantified for each cell type. The Wilcoxon rank-sum test (P < 0.05) was applied to compare the expression differences of these genes between the *Utf1* ^cKO^ and Ctrl groups. Cell types in which the biomarkers exhibited high overall biomarker abundance and showed significant differential expression between the two groups were defined as key cells. To characterize intercellular communication among annotated cell populations, cell-cell interaction analysis was performed on the *Utf1* ^cKO^ and Ctrl groups using the CellChat package (v 1.6.1) (Jin et al., 2025). Potential ligand-receptor pairs were predicted to evaluate signaling crosstalk between key cells and other cell types.

### 2.12 Functional enrichment and pseudotime trajectory analyses

To characterize the biological pathways and functions of the annotated cells, functional enrichment analysis was performed using the analyze_sc_clusters function in the “ReactomeGSA” package (v 1.14.0) (Griss et al., 2020) (P < 0.05). To further understand the differentiation process of key cells and the dynamic temporal trajectory of biomarker expression, key cells were re-clustered into distinct subclusters (resolution = 0.02) following the aforementioned method. Developmental trajectories of cell states were reconstructed using the “monocle” package (v 2.28.0) (Qiu et al., 2017), followed by pseudotime trajectory analysis. Additionally, the expression dynamics of biomarkers along the pseudotime axes were computationally profiled.

### 2.13 Generation of UTF1 conditional knockout mouse model and mating experiments

*Utf1* ^fl/+^ transgenic mice aged 6–8 weeks (purchased from GemPharmatech Co., Ltd., Jiangsu, China; strain catalog No. T037717) were intercrossed to generate *Utf1* ^fl/fl^ mice. Subsequently, *Utf1* ^fl/fl^ mice were crossed with *Vasa*-Cre tool mice (Lin et al., 2022) (originally described by Huijuan Lin et al., 2022) to ultimately generate *Utf1* ^fl/-^ *Vasa*Cre/+ mice, designated as *Utf1* ^cKO^ mice. Mouse genotypes were determined by nucleic acid electrophoresis of tail DNA (Supplementary Figure S1), and the PCR genotyping primers are listed in Supplementary Table S1. Male *Utf1* ^cKO^ mice at 60 days postpartum (dpp) were co-housed with wild-type male mice aged 6–8 weeks at a ratio of 1:3 overnight, and vaginal plugs were examined the following morning. The day of plug detection was designated as embryonic day 1 (1 dpc, days post coitum). All mice were housed in the Animal Experimental Center of our university under a 12-h light/dark cycle, with the specific pathogen-free facility maintained at a temperature of 21 °C ± 2 °C. The study was conducted in accordance with the revised Animals (Scientific Procedures) Act 1986 in the UK and Directive 2010/63/EU in Europe, and approved by the Laboratory Animal Welfare and Ethics Committee of Ningxia Medical University (Approval No. IACUC-NYLAC-2023-205).

### 2.14 Collection of mouse testes and sperm

Upon specimen collection, the bilateral testes were weighed, and the wet weights were recorded. The testicular index was calculated according to the following formula: Testicular index (%) = (average wet weight of bilateral testes (g)/body weight of mouse (g)) × 100%. Sperm samples preserved in a 37 °C incubator were placed onto glass slides and observed under a microscope. Computer-assisted sperm analysis (CASA) software was used to automatically analyze and generate reports on sperm count and motility. Testicular tissues were accurately weighed and homogenized in pre-cooled PBS at a ratio of 1:9, followed by centrifugation at 4 °C and 5,000 rpm for 10 min. The supernatant was collected, and serum testosterone levels were measured using a testosterone ELISA kit (Jiangsu Jingmei Biotechnology Co., Ltd.) according to the manufacturer’s instructions. Samples, standards, and reagents were added sequentially, followed by incubation, plate washing, and addition of chromogenic solution. After termination, absorbance was measured at 450 nm, and testosterone concentrations were calculated from the standard curve.

### 2.15 Hematoxylin and eosin (HE) staining and immunofluorescence

Slides were removed from the oven, deparaffinized in xylene, and progressively rehydrated through a descending ethanol gradient before rinsing under tap water. Hematoxylin staining was applied for 2 min, followed by tap water bluing. Sections were rapidly differentiated in 0.8% hydrochloric acid–alcohol, re-rinsed, counterstained with 0.5% eosin for 2 min, and dehydrated through an ascending ethanol series. After clearing in xylene, coverslips were affixed using neutral resin. Testicular morphology was subsequently examined under light microscopy. Immunofluorescence staining was performed as follows: tissue sections were deparaffinized, rehydrated, and incubated with 0.3% Triton X-100 in PBS at room temperature for 20 min. After PBS washes, excess liquid was removed, and 200 μL of primary antibody was added for overnight incubation at 4 °C. After PBS washes in the dark, 200 μL of fluorescent secondary antibody was added and incubated at 4 °C for 2 h. Sections were then washed with PBS three times (5 min each), mounted with DAPI-containing antifade mounting medium, air-dried in the dark, and observed and photographed under a fluorescence microscope. The UTF1 rabbit polyclonal antibody (Ab24273, Abcam) and antifade mounting medium with DAPI (S2110, Solarbio) were used.

### 2.16 Immunohistochemistry

Sections were deparaffinized in xylene and rehydrated through a graded ethanol series. Antigen retrieval was conducted by immersing slides in citrate buffer and microwaving for 20 min, followed by passive cooling to room temperature. Endogenous peroxidase activity was blocked by incubating sections in a humidified chamber with peroxidase blocker for 10 min at room temperature, after which slides were rinsed three times in PBS (3 min each). Primary antibodies were applied in volumes proportional to tissue size and incubated overnight at 4 °C. After warming to room temperature, sections were rinsed three times in PBS, treated with signal enhancement reagent at 37 °C for 20 min, and rinsed again. Enhanced enzyme-labeled goat anti-mouse/rabbit IgG polymer was then applied at 37 °C for 20 min, followed by three PBS washes. Freshly prepared DAB chromogen was dispensed, and color development was monitored microscopically for 3–8 min before termination with tap water. Sections were counterstained with hematoxylin for 20 s, differentiated, blued, cleared in xylene, and mounted with neutral resin.

### 2.17 TUNEL assay

Testicular tissue sections were cleared in xylene I and II for 10 min each, rehydrated through anhydrous ethanol, 90% ethanol, and 70% ethanol for 3 min each, and rinsed with ultrapure water for 2 min. Sections were then incubated with 20 μg/mL DNase-free proteinase K in a humidified chamber at 25 °C for 20 min and washed three times with PBS. The TUNEL reaction mixture containing TdT was added, and sections were incubated in the dark at 37 °C for 1 h, followed by three PBS washes. DAPI was applied for counterstaining at room temperature in the dark for 5 min, followed by three PBS washes. Sections were mounted with antifade mounting medium and observed under a fluorescence microscope.

### 2.18 Statistical analysis

All bioinformatics pipelines were executed in R (v 4.3.3), with between-group comparisons evaluated by the Wilcoxon rank-sum test (P < 0.05). Experimental data were analyzed using SPSS 22.0 and GraphPad Prism 8.0. Quantitative variables are expressed as mean ± standard deviation. Two-group comparisons were made using the two-sample Student’s t-test, whereas one-way ANOVA was applied for multi-group comparisons. A P-value below 0.05 was considered statistically significant throughout.

## 3 Results

### 3.1 Single-cell sequencing-based characterization of cellular subsets and hdWGCNA

After rigorous quality control (Supplementary Figure S2), the single-cell transcriptomic profiling was performed on 49,901 cells and 26,608 genes obtained from the *Utf1* ^cKO^ and Ctrl groups. The top 2,000 HVGs were selected (Supplementary Figure S3) and PCA based on variance-stabilized HVGs identified 20 significant PCs (Supplementary Figure S4). Uniform manifold approximation and projection (UMAP) visualization resolved 10 transcriptionally distinct cell clusters (Supplementary Figure S5). Cell type annotation was conducted using canonical marker genes exhibiting cluster-specific expression patterns (Figures 1A,B). This analysis identified eight major cell populations (Figure 1C): spermatogonia, epithelial cells, spermatids, Sertoli cells, Leydig cells, endothelial cells, macrophages, and peritubular cells. Cells lacking distinct marker gene signatures and displaying highly variable, noisy expression profiles were defined as “Others”. From the eight annotated cell populations, spermatogonia, Sertoli cells, and spermatids—cell types critically involved in spermatogenesis—were selected for hdWGCNA to identify spermatogenesis-related genes. The optimal soft-thresholding power for network construction was determined to be seven (R^2^ = 0.92, mean connectivity <200) (Figure 1D). A total of three gene co-expression modules were identified after excluding the unassigned grey module (Figure 1E). Genes in these three modules were markedly enriched in spermatogenic cell types (spermatogonia, Sertoli cells, and spermatids) (Figure 1F). Subsequently, 300 KMRGs were identified, and 240 human orthologs of these KMRGs (defined as spermatogenesis-related genes) were retrieved for downstream analysis.

**FIGURE 1:**
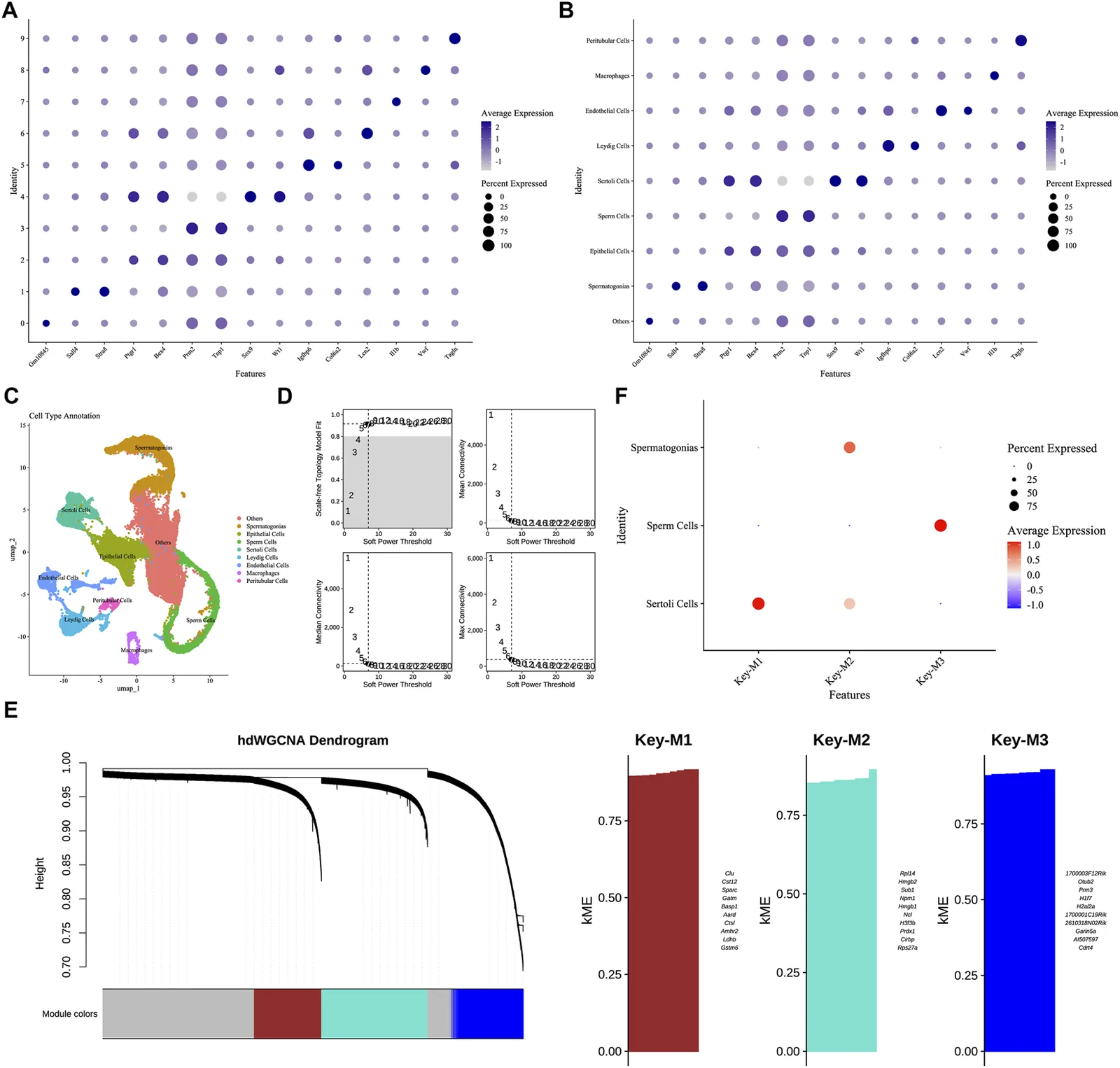
Single-cell transcriptomic profiling and hdWGCNA analysis of mouse testicular tissues. **(A,B)** Expression patterns of canonical marker genes across clusters for cell-type annotation before and after refinement. **(C)** UMAP plot showing the distribution of eight major annotated cell populations and “Others” cells. **(D)** Selection of the optimal soft-thresholding power for hdWGCNA network construction. **(E)** Dendrogram of gene co-expression modules identified by hdWGCNA. **(F)** Bubble plot showing the percentages and mean expression levels of genes from three key modules across different cell types closely associated with spermatogenesis.

### 3.2 Screening for candidate genes and underlying biological mechanisms

Differential expression analysis of NOA *versus* control testicular tissues within the GSE145467 dataset uncovered 4,688 DEGs (P.adj <0.05, |log_2_FC| > 1), of which 1,550 were upregulated and 3,138 downregulated in NOA (Figures 2A,B). Overlaying this DEG list against the 240 spermatogenesis-related genes produced 73 candidate genes (Figure 2C). GO enrichment yielded 153 significantly enriched terms (P < 0.05), covering biological processes such as spermatid differentiation, sperm motility, flagellum assembly, sperm DNA condensation, and the acrosome reaction (Figure 2D; Supplementary Table S2). At the pathway level, KEGG analysis highlighted 21 significantly enriched pathways (P < 0.05), among them glutathione metabolism, reactive oxygen species response, glycolysis, and fatty acid degradation (Supplementary Table S3). PPI network construction further demonstrated intricate functional interconnections among the 73 candidate genes (Figure 2E). Taken together, these observations indicated that the candidate genes were deeply implicated in NOA pathogenesis and spermatogenic dysfunction, with their aberrant expression likely contributing to disease onset.

**FIGURE 2:**
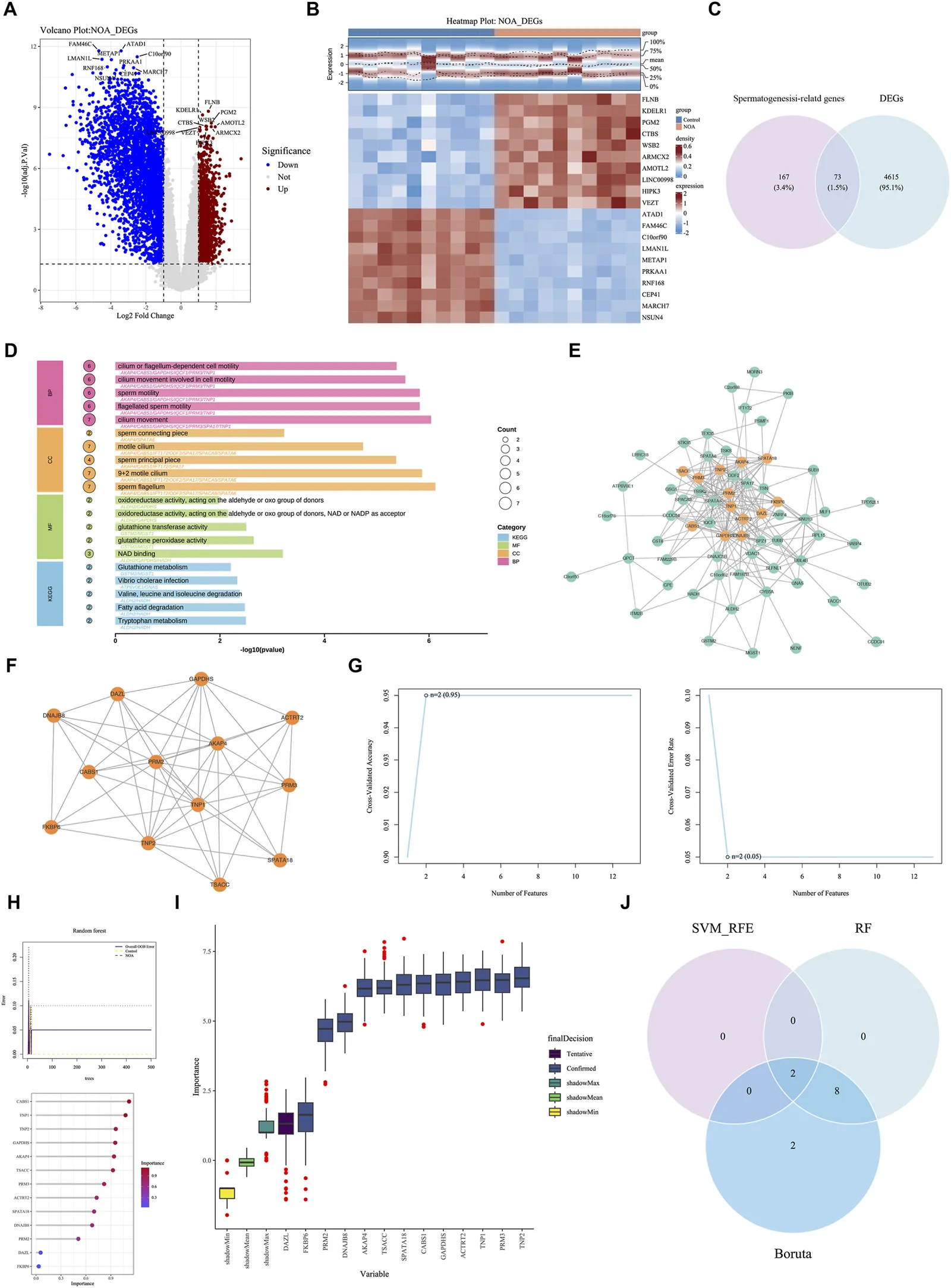
Screening of candidate biomarkers and functional analysis. **(A)** Volcano plot of differentially expressed genes (DEGs) between non-obstructive azoospermia (NOA) and control tissues. **(B)** Heatmap of the top 20 DEGs illustrating distinct expression patterns between NOA and control samples. **(C)** Venn diagram showing the intersection between DEGs and spermatogenesis-related genes. **(D)** Gene Ontology (GO) and Kyoto Encyclopedia of Genes and Genomes (KEGG) enrichment analyses of the candidate genes. **(E)** Protein–protein interaction (PPI) network depicting functional associations among the candidate genes. **(F)** Key subnetwork with the highest Molecular Complex Detection (MCODE) score extracted from the PPI network. **(G)** Classification accuracy of support vector machine–recursive feature elimination (SVM-RFE) with varying numbers of retained genes. **(H)** Random Forest analysis ranking genes according to the Mean Decrease Gini coefficient. **(I)** Feature importance plot generated using the Boruta algorithm. **(J)** Venn diagram showing the intersection of genes screened using the three machine-learning algorithms, yielding candidate biomarkers.

### 3.3 Identification of biomarkers and performance evaluation of the nomogram

Based on the aforementioned PPI network, four subnetworks were further identified, among which the key subnetwork with the highest score (8.33) yielded 13 candidate genes (Figure 2F). Three distinct machine learning algorithms were employed for further screening. SVM-RFE achieved optimal classification performance, with the lowest cross-validation error (0.05) and the highest accuracy (0.95), retaining two genes: ACTRT2 and AKAP4 (Figure 2G). Concurrently, the RF analysis ranked genes based on the Mean Decrease Gini coefficient, retaining the top 10 genes (Figure 2H). The Boruta algorithm identified 12 genes labeled as “Confirmed” features (Figure 2I). The intersection of genes selected by the three algorithms yielded two candidate genes: ACTRT2 and AKAP4 (Figure 2J). Subsequent expression analysis revealed that ACTRT2 and AKAP4 were significantly downregulated in NOA samples compared with controls in both the GSE145467 and GSE45887 datasets (P < 0.05) (Figures 3A,B). To further assess model generalizability, the three models constructed in the training cohort were applied to the independent validation cohort GSE45887. In the training cohort, all three models performed well (AUC: Boruta = 1.000, Random Forest = 1.000, SVM-RFE = 0.920). In the validation cohort, however, performance was limited (Boruta = 0.750, Random Forest = 0.750, SVM-RFE = 0.125), and the models tended to classify all validation samples as controls, indicating insufficient generalizability. This may be attributable to the small sample size and marked class imbalance of the validation cohort (16 NOA vs. 4 controls), as well as cross-platform and cross-cohort batch effects (Supplementary Figures S6A–D). In addition, UTF1 expression was validated by the Wilcoxon rank-sum test in both cohorts. UTF1 was significantly upregulated in NOA samples in the GSE145467 training cohort (P < 0.05), whereas no significant difference was observed in the GSE45887 validation cohort (P > 0.05), with an opposite trend (Supplementary Figures S6E and F). A nomogram model was constructed to predict NOA risk based on the expression levels of ACTRT2 and AKAP4 (Figure 3C). In this model, each biomarker was assigned a score proportional to its expression level, with the sum of scores representing the total risk points. For instance, a total score of 149 corresponded to a 94.6% predicted probability of NOA, demonstrating the model’s utility in individualized risk stratification. The calibration curve was plotted to evaluate the agreement between the predicted probabilities and actual clinical outcomes (Figure 3D). The HL test confirmed good model fit (P = 0.911). These results confirmed that ACTRT2 and AKAP4 were reliable biomarkers for NOA, and the constructed nomogram had good predictive performance for NOA risk.

**FIGURE 3:**
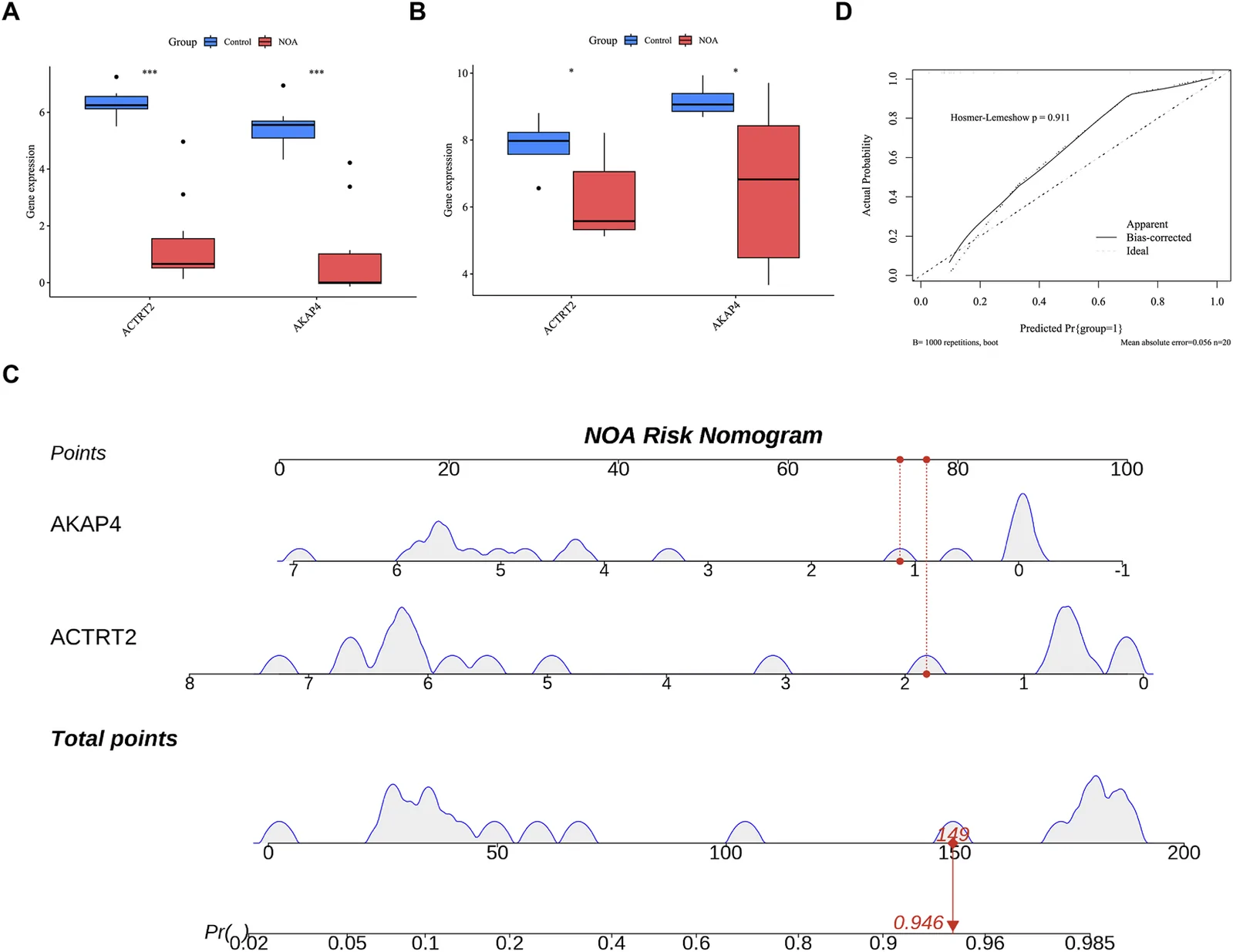
Expression validation and nomogram construction for the biomarkers. **(A,B)** Boxplots demonstrating the differential expression of candidate biomarkers between NOA and control samples in the GSE145467 and GSE45887 datasets. **(C)** Nomogram for predicting NOA risk based on ACTRT2 and AKAP4 expression. **(D)** Calibration curve of the nomogram.

### 3.4 Chromosomal and subcellular localization and pathway enrichment of ACTRT2 and AKAP4

Chromosomal mapping revealed ACTRT2 was located on chromosome 1 and AKAP4 on chromosome X (Figure 4A). Subcellular localization prediction indicated that ACTRT2 predominantly resided in the cytoskeleton, cytoplasm, followed by the nucleus, endoplasmic reticulum, mitochondria, and plasma membrane (Figure 4B). In contrast, AKAP4 was primarily localized to the nucleus, with secondary distribution in the cytoplasm, cytoskeleton, mitochondria, plasma membrane, Golgi apparatus, lysosome, endosome, endoplasmic reticulum, and extracellular region. Functional enrichment analysis was further performed to explore the biological pathways associated with ACTRT2 and AKAP4, respectively (Figure 4C; Supplementary Tables S4 and S5). For ACTRT2, the 52 significantly enriched pathways were mainly involved in microtubule dynamics and cytoskeleton remodeling. For AKAP4, the 59 enriched pathways predominantly focused on flagellum assembly and meiotic spindle regulation. Notably, 51 pathways were commonly shared between ACTRT2 and AKAP4, including core modules governing microtubule nucleation, depolymerization, and kinetochore-microtubule attachment. In summary, these results demonstrated that ACTRT2 and AKAP4 exhibited distinct chromosomal localization but overlapping subcellular distribution and functional pathways, which were closely associated with NOA pathogenesis and normal spermatogenesis.

**FIGURE 4:**
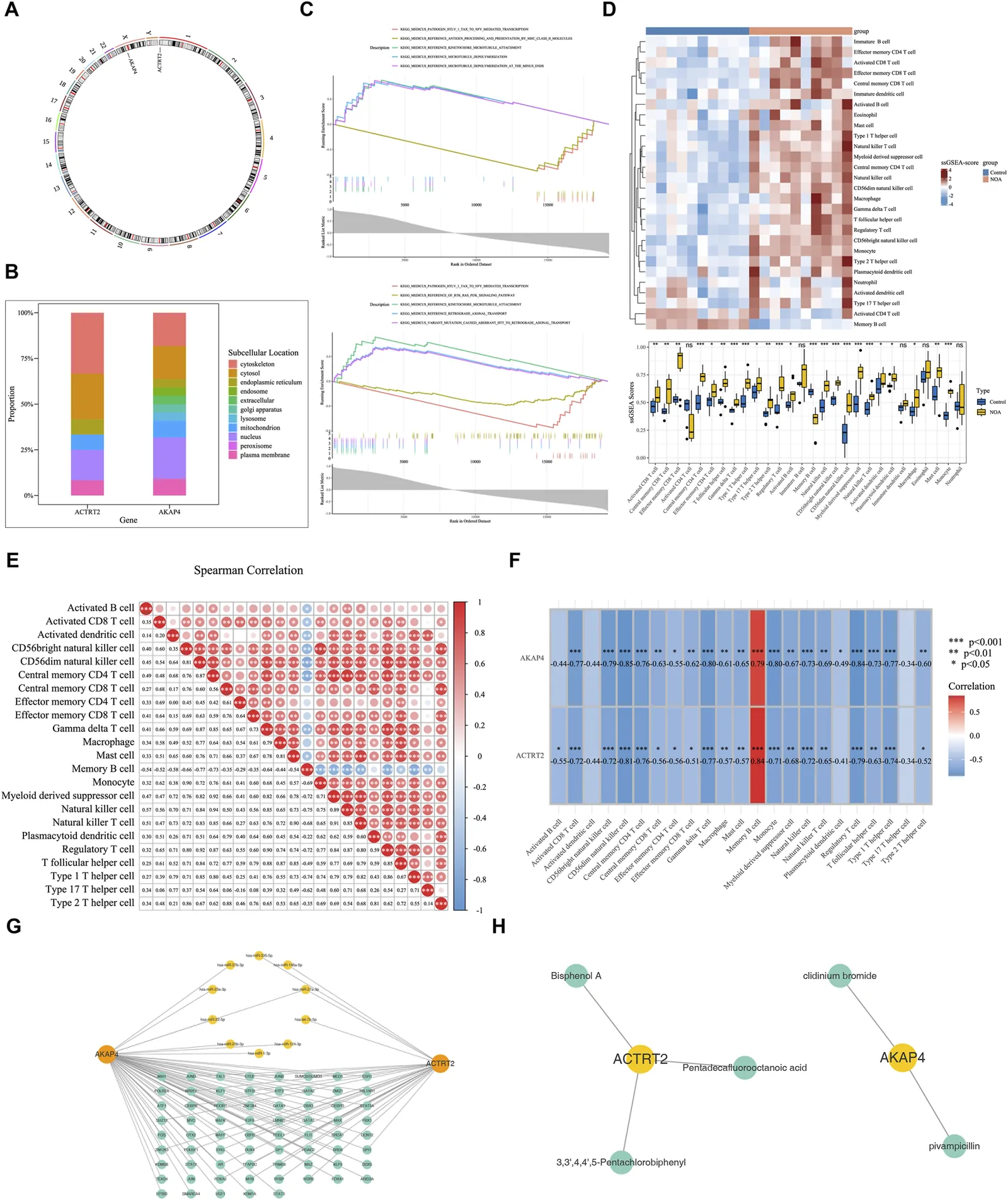
Genomic and subcellular localization, functional enrichment, immune infiltration, regulatory mechanisms, and drug prediction for the biomarkers. **(A,B)** Chromosomal mapping and subcellular localization prediction of ACTRT2 and AKAP4. **(C)** Representative gene set enrichment analysis (GSEA) results for ACTRT2 and AKAP4. **(D)** Heatmap showing the proportions of 28 immune cell subsets in NOA and control samples and the differentially infiltrated immune cells between the two groups. **(E)** Correlation heatmap of differentially infiltrated immune cells. **(F)** Correlation heatmap between ACTRT2/AKAP4 expression and differentially infiltrated immune cells. **(G)** miRNA–mRNA–transcription factor regulatory network of ACTRT2 and AKAP4. **(H)** Drug–gene interaction network of ACTRT2 and AKAP4.

### 3.5 Immune cell infiltration and its correlation with biomarkers

Profiling of 28 immune cell subsets revealed significant infiltration differences in 23 populations between NOA and control groups (P < 0.05) (Figure 4D). Of these, 22 subsets were elevated in NOA—prominently including macrophages, regulatory T cells (Tregs), myeloid-derived suppressor cells (MDSCs), and mast cells—whereas memory B cells were the sole population markedly reduced. Pairwise correlation mapping among the differentially infiltrated subsets uncovered extensive co-regulatory networks (Figure 4E). Tregs demonstrated the most pronounced positive co-variation with gamma delta T cells (cor = 0.90, P < 0.001), CD56dim natural killer (NK) cells (cor = 0.92, P < 0.001), and MDSCs (cor = 0.84, P < 0.001). Macrophages co-varied strongly with T follicular helper cells (cor = 0.87, P < 0.001) and mast cells (cor = 0.81, P < 0.001). Memory B cells, conversely, showed broad negative co-variation with most upregulated immune populations, most markedly with NK cells (cor = −0.75, P < 0.001) and CD56dim NK cells (cor = −0.77, P < 0.001). Biomarker–immune cell correlation analysis further demonstrated that both ACTRT2 and AKAP4 were significantly inversely associated with the majority of T-cell subsets (including Tregs), NK cells, MDSCs, macrophages, mast cells, and monocytes (cor < −0.5, P < 0.01) (Figure 4F), while displaying prominent positive associations with memory B cells (ACTRT2: cor = 0.84, P < 0.001; AKAP4: cor = 0.79, P < 0.001). These data collectively delineated the immune infiltration landscape of NOA and highlighted its bidirectional coupling with ACTRT2 and AKAP4 expression.

### 3.6 Prediction of potential regulatory mechanisms and drugs

To elucidate the transcriptional regulation of ACTRT2 and AKAP4, a miRNA-mRNA-TF interaction network was constructed (Figure 4G). Four miRNAs were predicted to target ACTRT2, whereas seven miRNAs targeted AKAP4. Notably, hsa-miR-27a-3p was identified as a shared regulator of both genes. In addition, 24 TFs were predicted to regulate ACTRT2, and 55 TFs regulated AKAP4. Among these, 10 TFs commonly regulated both genes, including AR, FOXA2, SPI1, and JUND. Drug-gene interaction analysis further identified three potential compounds targeting ACTRT2, namely, bisphenol A, 3,3′,4,4′,5-pentachlorobiphenyl, and pentadecafluorooctanoic acid (Figure 4H). Two drugs were predicted to target AKAP4, including clidinium bromide and pivampicillin. Collectively, these findings delineated the transcriptional regulatory networks of ACTRT2 and AKAP4, offering potential mechanistic targets and candidate agents for NOA.

### 3.7 Identification of key cells and intercellular communication networks

As shown in Figure 5A, ACTRT2 and AKAP4 were highly expressed in sperm cells, and ACTRT2 exhibited significant intergroup differences in this cell population between the *Utf1* ^cKO^ and Ctrl groups (Figure 5B). Based on these findings, sperm cells were selected as the key cell population for subsequent analyses. Cell-cell communication analysis revealed that the *Utf1* ^cKO^ group exhibited a marked increase in both the number and strength of intercellular interactions compared with controls, particularly among Sertoli cells, spermatogenic cells, and immune cells (Figures 5C,D). Notably, sperm cells and endothelial cells displayed extensive incoming signaling interactions with multiple cell types, including peritubular cells. Analysis of sperm cell-centered ligand-receptor interactions further demonstrated that sperm cells initiated distinct signaling programs in the Ctrl and *Utf1* ^cKO^ groups (Figure 5E). In the Ctrl group, sperm cells engaged in relatively limited crosstalk, primarily *via* the App-Cd74 axis with macrophages, as well as Cadm1-Cadm1-mediated adhesion with Sertoli cells and spermatogonia. In striking contrast, the *Utf1* ^cKO^ group showed expanded sperm cell communication, with *de novo* interactions involving peritubular cells, endothelial cells, and epithelial cells, mediated by ligand-receptor pairs such as Dll3-Notch1/3, Grn-Sort1, and Cadm1-Cadm1. Collectively, these results indicated that sperm cell-centered communication was globally rewired in the NOA-like testicular microenvironment.

**FIGURE 5:**
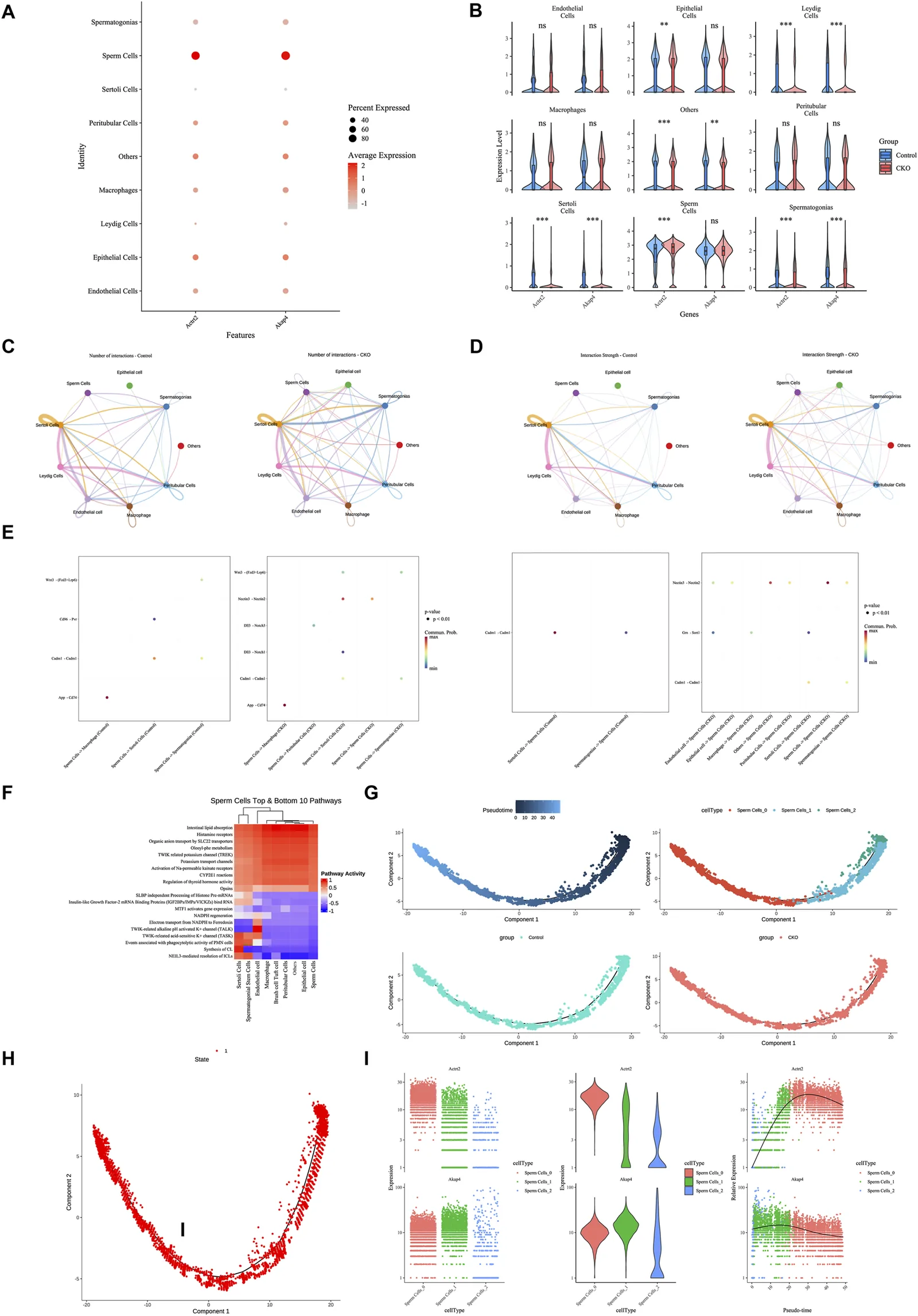
Key cell identification, cell–cell communication, functional pathways, and differentiation trajectory analysis. **(A)** Expression distribution of ACTRT2 and AKAP4 across annotated cell types. **(B)** Differences in ACTRT2 and AKAP4 expression in sperm cells between the Utf1cKO and control (Ctrl) groups. **(C,D)** Cell–cell communication analysis showing the number and strength of intercellular interactions in the Ctrl and Utf1cKO groups. **(E)** Dot plot of sperm cell-centered ligand–receptor interactions in the Ctrl and Utf1cKO groups. **(F)** Pathway activity heatmap showing the top and bottom 10 pathways in sperm cells across annotated cell populations. **(G–H)** Pseudotime trajectory analysis of sperm cell differentiation. **(I)** Stage-specific expression dynamics of ACTRT2 and AKAP4 along the pseudotime axis.

### 3.8 Functional pathways and differentiation trajectory of sperm cells

Pathway activity analysis was performed to characterize the functional landscape of the annotated testicular cell populations, with a focus on sperm cells (Figure 5F). Sperm cells exhibited a distinct pathway signature characterized by highly activated metabolic and ion channel-related pathways, including intestinal lipid absorption, histamine receptor signaling, organic anion transport by SLC22 transporters, and potassium transport channels. In contrast, pathways related to RNA processing, immune function, and oxidative stress resolution were strongly suppressed in sperm cells, such as SLBP-independent processing of histone pre-mRNAs, insulin-like growth factor-2 mRNA binding proteins (IGF2BPs/IMPs/VICKZs) binding to RNA, and NEIL3-mediated resolution of ICLs. These findings highlighted the specialized functional role of sperm cells in spermatogenesis. Subclustering of sperm cells resolved these cells into three transcriptionally distinct subpopulations (Supplementary Figure S7). Pseudotime trajectory analysis based on transcriptional profiling identified a single cellular continuum corresponding to progressive differentiation stages (Figures 5G,H). Mapping of the three sperm cell subclusters to these states revealed their sequential distribution across differentiation stages. Additionally, compared with the Ctrl group, the *Utf1* ^cKO^ group exhibited fewer early-stage and more late-stage differentiated sperm cells. Stage-specific expression analysis of the biomarkers along the pseudotime axis and across cellular states revealed that ACTRT2 expression was markedly upregulated in the early differentiation stage, peaked at the mid-stage, and then declined gradually, while AKAP4 expression increased slowly to a peak in the early-to-mid stage, followed by a sustained gradual decrease (Figure 5I). These results demonstrated that *Utf1* ^cKO^ -induced NOA was associated with disrupted spermatogenic differentiation dynamics and stage-specific dysregulation of ACTRT2 and AKAP4.

### 3.9 Utf1 deficiency leads to testicular development impairment and reduced sperm count and motility

To further validate whether *Utf1* ^cKO^ mice could serve as a suitable model for subsequent experiments, we evaluated UTF1 protein levels in the testicular tissue of *Utf1* ^cKO^ mice using immunohistochemistry (IHC) and Western blotting. UTF1 expression was assessed at two critical developmental time points: 8 dpp and 35 dpp. Quantitative IHC analysis revealed that, compared with littermate control mice (control males: *Utf1* ^fl/+^, *Utf1* ^fl/-^, *Utf1* ^fl/+^ *Vasa*-Cre), the UTF1 protein signal intensity in the testicular tissue of *Utf1* ^cKO^ mice was significantly decreased at both 8 dpp and 35 dpp (****, P < 0.0001) (Supplementary Figures S8A and B). To validate these findings at the total protein level, we performed Western blotting on testicular tissue from 8 dpp mice. The results consistently demonstrated that UTF1 protein levels in the testis of *Utf1* ^cKO^ mice were significantly reduced compared with those in littermate controls (***, P < 0.001) (Supplementary Figures S8C).

Subsequently, to investigate the effects of UTF1 deficiency on testicular development and spermatogenesis, we weighed the testes of *Utf1* ^cKO^ mice and their littermate controls at 35 dpp and assessed sperm count and motility in the cauda epididymis. Compared with littermate controls, *Utf1* ^cKO^ mice exhibited varying degrees of reduced body size (Figure 6A). The testis-to-body weight ratio was decreased in *Utf1* ^cKO^ mice compared with littermate controls (Figure 6C), with a significant reduction observed specifically at 35 dpp (***, P < 0.001) (Figure 6B). Sperm count and motility were assessed by computer-assisted sperm analysis (CASA) of cauda epididymal sperm. At 35 dpp, sperm count was significantly decreased in Utf1cKO mice compared with littermate controls (****P < 0.0001) (Figure 6D), and sperm motility was also significantly reduced (***P < 0.001) The detailed CASA results are provided in Supplementary Table S6. To further evaluate the effect of UTF1 deficiency on male reproductive endocrine function, serum testosterone levels were measured in mice at 35 dpp. As shown in Supplementary Figures S8D, serum testosterone levels were significantly lower in the Utf1cKO group than in the Ctrl group (***P < 0.001). These results indicated that UTF1 deficiency was accompanied by a marked decrease in testosterone levels at 35 dpp, suggesting a potential impact on testicular endocrine function.

**FIGURE 6:**
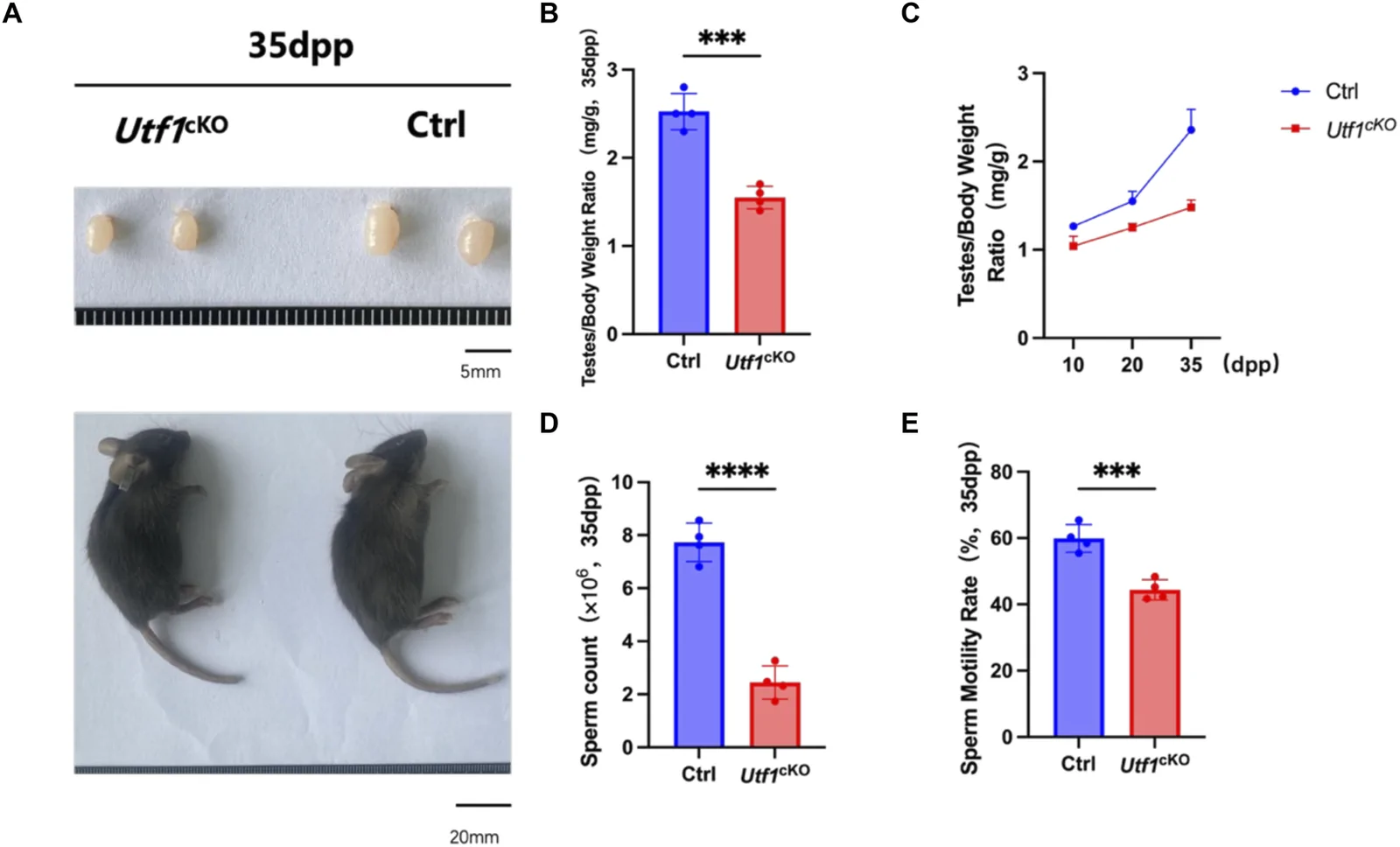
Utf1 knockout causes testicular hypoplasia and impaired spermatogenesis. **(A)** Mice and testes at 35 days postpartum (dpp). **(B)** Testis-to-body weight ratio in mice at 35 dpp. **(C)** Changes in the testis-to-body weight ratio at 10, 20, and 35 dpp. **(D)** Sperm count in mice at 35 dpp. **(E)** Sperm motility in mice at 35 dpp. ***, P < 0.001; ****, P < 0.0001.

### 3.10 Utf1 deficiency leads to male infertility in mice

To assess whether *Utf1* knockout affects male fertility, we performed mating experiments using mice of different genotypes at 60 dpp at a ratio of 1:3. Compared with wild-type mating pairs and pairs consisting of *Utf1* ^flox/+^ *Vasa*Cre^+^ male mice with wild-type female mice, the number of vaginal plug detections and total offspring were reduced in pairs consisting of *Utf1* ^cKO^ male mice with wild-type female mice (Table 1). Statistical analysis of the average litter size revealed that *Utf1* ^cKO^ male mice mated with wild-type female mice produced a significantly lower average litter size compared with wild-type mating pairs and pairs consisting of *Utf1* ^flox/+^ *Vasa*Cre^+^ male mice with wild-type female mice (***, P < 0.001) (Figure 7).

**TABLE 1:** Mating experiments using mice of different genotypes.

No.	Genotype of mating experiments	Number of cages	Number of vaginal plugs	Total Little size	Average litter size
1	WT(M) × WT(F)	4	12	29	7.2
2	*Utf1* ^flox/+^VasaCre+(M) × WT(F)	4	12	28	7
3	*Utf1* ^cKO^(M) × WT(F)	3	6	6	2

**FIGURE 7:**
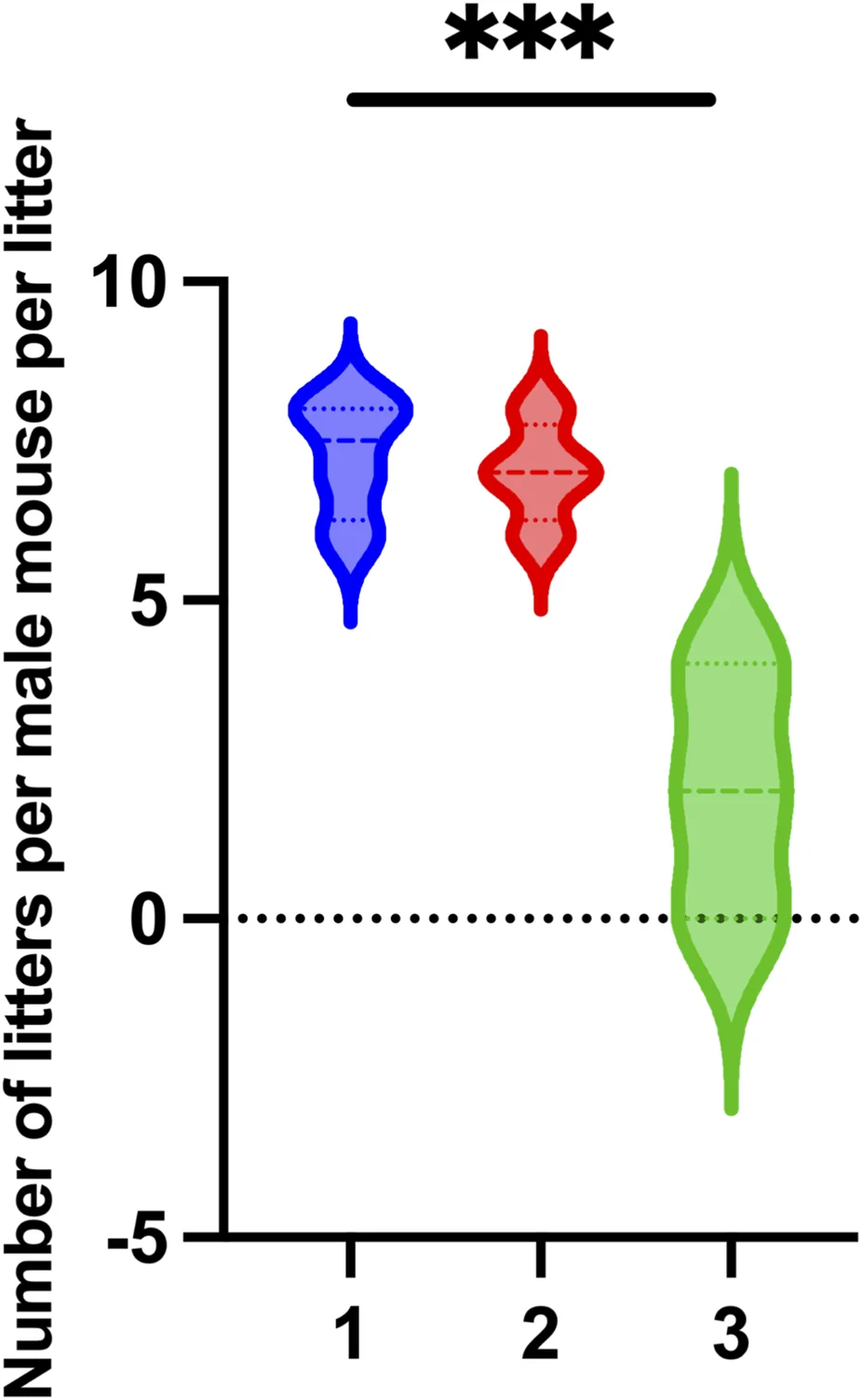
Average litter size per cage after mating mice of different genotypes. Blue, wild-type male × wild-type female; red, Utf1flox/+ VasaCre + male × wild-type female; green, Utf1cKO male × wild-type female. ***, P < 0.001.

### 3.11 Utf1 deficiency leads to dysfunction in the first wave of spermatogenesis and loss of germ cells at various stages in seminiferous tubules

To systematically investigate the first wave of spermatogenesis in male mice with conditional knockout of UTF1 (*Utf1* ^cKO^), we performed H&E staining and histomorphological analysis on testicular tissues from *Utf1* ^cKO^ male mice at 10 dpp, 20 dpp, and 35 dpp, as well as from littermate control mice at 35 dpp. The results revealed that, compared with the orderly and intact seminiferous epithelial structure observed in control mice at corresponding time points, *Utf1* ^cKO^ mice exhibited marked abnormalities in cellular composition within the seminiferous tubules. Specifically, a reduction in the number of spermatogonia was observed as early as 10 dpp. By 20 dpp and 35 dpp, varying degrees of depletion of spermatocytes and round spermatids were evident, accompanied by disorganized cellular layering within the seminiferous tubules, ultimately leading to the absence of mature spermatozoa in the lumina (Figures 8A,B). In addition, immunofluorescence staining of wild-type mouse testes showed that UTF1 expression exhibited a clear spatiotemporal pattern: at 8 dpp, UTF1 was mainly localized in the nuclei of spermatogonia, whereas at 35 dpp, its expression was predominantly distributed in the nuclei of round spermatids. These findings further supported the stage-specific role of UTF1 during spermatogenesis and provided morphological evidence for its function in specific germ cell types (Figure 8C).

**FIGURE 8:**
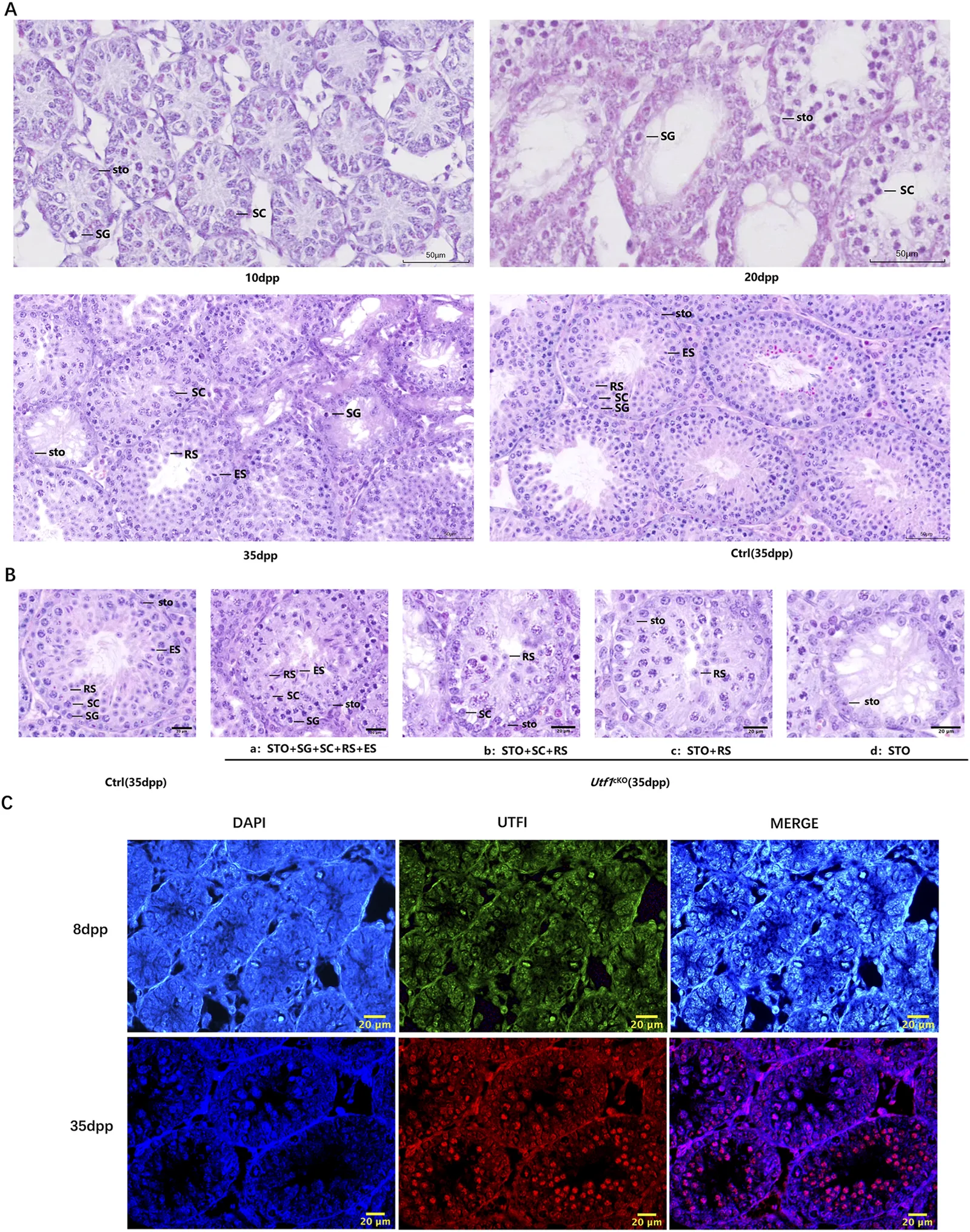
Utf1 deficiency disrupts the first wave of spermatogenesis and causes germ-cell loss in seminiferous tubules. **(A)** Morphology of seminiferous tubules in Utf1cKO male mice at 10, 20, and 35 dpp and in littermate control mice at 35 dpp. Scale bar, 50 μm. **(B)** Morphology of seminiferous tubules and loss of germ cells at different developmental stages in Utf1cKO male mice and littermate controls at 35 dpp. Scale bar, 20 μm. **(C)** Distribution of UTF1 expression in the testes of wild-type mice at 8 dpp and 35 dpp. Scale bar, 20 μm. SG, spermatogonia; SC, spermatocyte; RS, round spermatid; ES, elongated spermatid; STO, Sertoli cell.

### 3.12 Utf1 deficiency reduces proliferative capacity of testicular cells in Utf1cKO male mice at 35 dpp

To further investigate the causes of varying degrees of germ cell depletion within the seminiferous tubules of *Utf1* ^cKO^ male mice at 35 dpp, we assessed two proliferation markers, PCNA and MKi67, using immunohistochemistry. The results showed that PCNA expression was significantly decreased in testicular cells of *Utf1* ^cKO^ male mice compared with littermate controls (**, *P* < 0.01), and MKi67 expression was also significantly decreased (***, *P* < 0.001). These findings suggest that the proliferative capacity of germ cells is reduced in the testes of *Utf1* ^cKO^ male mice compared with littermate controls (Figure 9).

**FIGURE 9:**
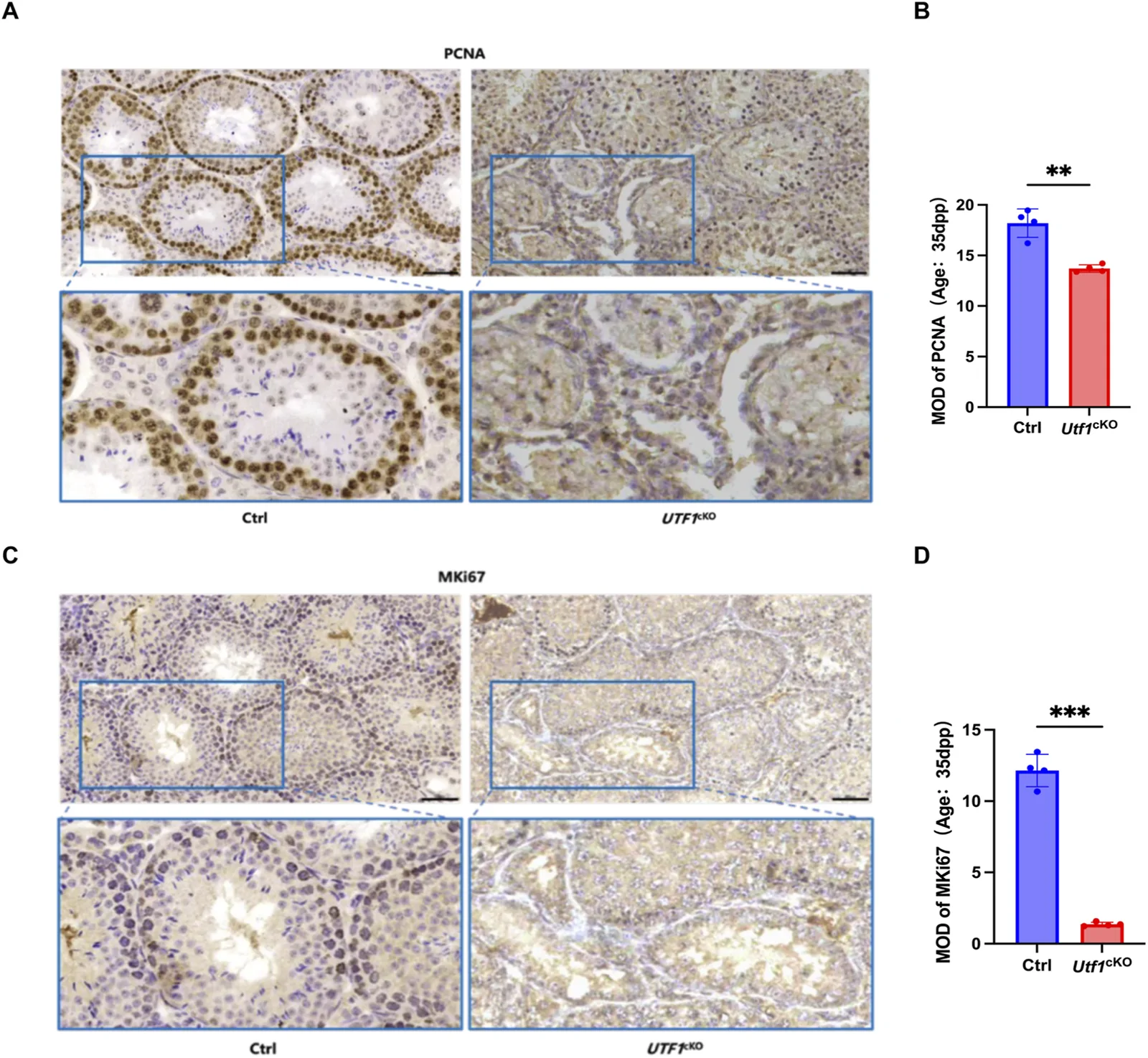
Utf1 deficiency reduces the proliferative capacity of testicular germ cells in Utf1cKO male mice at 35 dpp. **(A,B)** Immunohistochemical staining of proliferating cell nuclear antigen (PCNA) in the testes of Utf1cKO mice and littermate controls at 35 dpp and quantification of its expression. Scale bar, 50 μm **, P < 0.01. **(C,D)** Immunohistochemical staining of MKI67 in the testes of Utf1cKO mice and littermate controls at 35 dpp and quantification of its expression. Scale bar, 50 μm ***, P < 0.001.

### 3.13 TUNEL staining reveals increased germ cell apoptosis in Utf1cKO testes

To determine whether germ cell loss in Utf1cKO testes was associated with increased apoptosis, TUNEL staining was performed at 35 dpp. TUNEL-positive cells were markedly more abundant in the seminiferous tubules of Utf1cKO mice than in littermate controls. Quantitative analysis showed that the TUNEL-positive signal intensity was significantly higher in Utf1cKO testes than in controls (P < 0.01), indicating that enhanced germ cell apoptosis contributed to the progressive loss of germ cells following Utf1 deficiency (Figure 10).

**FIGURE 10:**
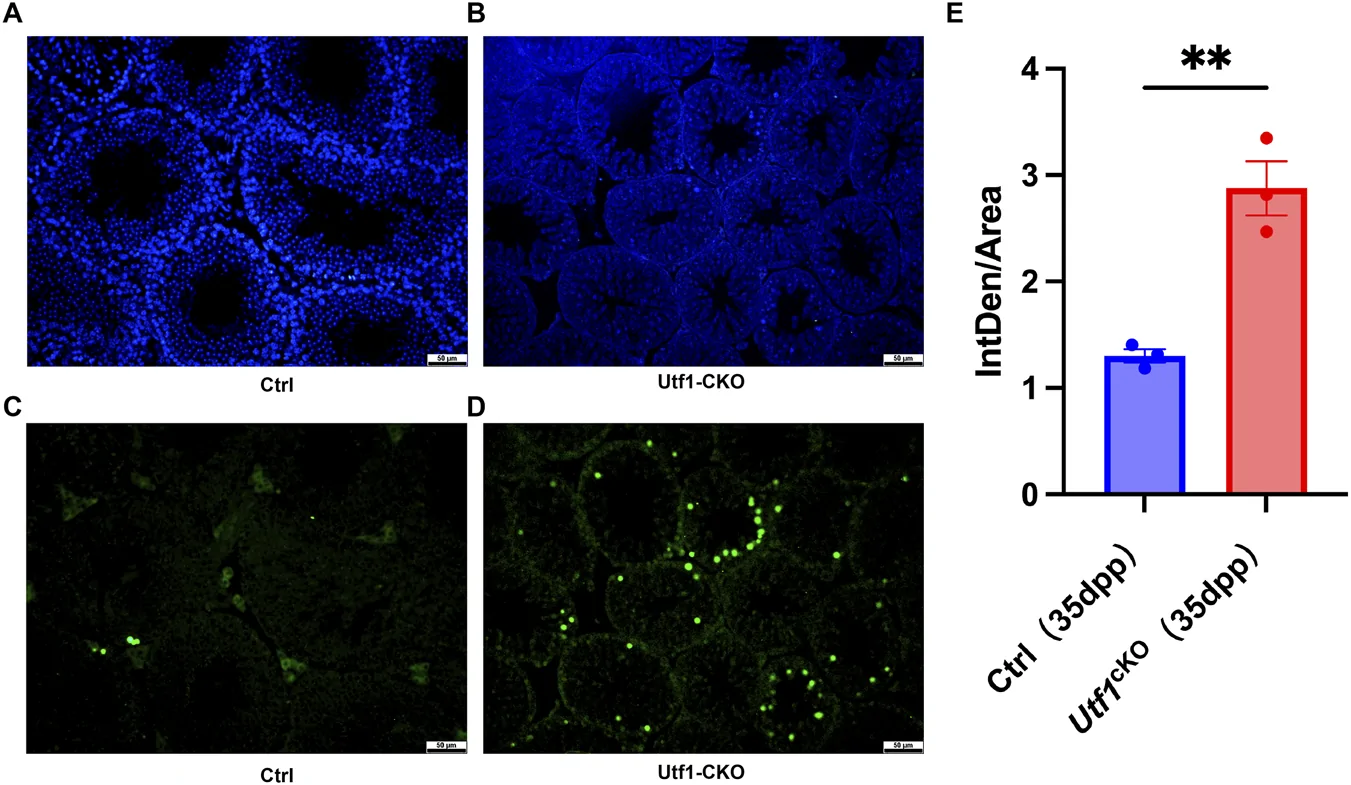
TUNEL staining reveals increased germ cell apoptosis in Utf1cKO testes at 35 dpp. **(A,B)** Representative DAPI staining of testicular sections from Ctrl **(A)** and Utf1cKO **(B)** mice at 35 dpp. Scale bar, 50 μm. **(C,D)** Representative TUNEL staining of testicular sections from Ctrl (C) and Utf1cKO **(D)** mice at 35 dpp. Scale bar, 50 μm. **(E)** Quantitative analysis of TUNEL-positive signal intensity (IntDen/Area) in Ctrl and Utf1cKO testes. Data are presented as mean ± SD. **P < 0.01.

## 4 Discussion

Recent studies indicate that NOA is not caused by a single germ cell defect, but rather results from the combined imbalance of spermatogonial maintenance, Sertoli cell function, local immune status, and intercellular communication, highlighting its marked heterogeneity (Piechka et al., 2024). Meanwhile, UTF1, as a key marker and potential regulator of undifferentiated spermatogonia, exhibits reduced expression and abnormal localization in NOA testes, suggesting its involvement in the disrupted spermatogenic program (Roshan et al., 2024). Against this backdrop, our integrated analysis of *Utf1* ^cKO^ mouse single-cell transcriptomic data and human NOA bulk data revealed that ACTRT2 and AKAP4 are consistently downregulated and associated with spermatogenic defects, immune imbalance, and disrupted cell communication, while phenotypic evidence further supports a critical role of UTF1 in maintaining normal spermatogenesis.

This study did not merely focus on whether UTF1 deficiency affects spermatogenesis; instead, it further contextualized UTF1-related abnormalities within the highly heterogeneous framework of NOA. By integrating human NOA bulk transcriptomic data with single-cell transcriptomic profiles from *Utf1* ^cKO^ mouse testes, we identified ACTRT2 and AKAP4 as two consistently downregulated potential biomarkers within spermatogenesis-related modules. These genes were further linked to abnormal immune infiltration, remodeling of spermatocyte-centered intercellular communication, and disrupted differentiation trajectories, suggesting that UTF1-associated spermatogenic defects are not simply due to reduced germ cell numbers, but are more likely part of a continuous process involving germ cell program imbalance, microenvironmental remodeling, and functional exhaustion. This is consistent with recent single-cell studies demonstrating that the key pathological feature of NOA is not the impairment of a single cell population, but rather a systemic imbalance involving spermatogonia, Sertoli cells, peritubular cells, immune cells, and vascular-associated cells (Jiang et al., 2025). Despite these advances, current clinical stratification and prediction of sperm retrieval outcomes in NOA still rely heavily on limited histological and empirical indicators. Therefore, a molecular biomarker framework that integrates both pathological mechanisms and clinical predictive value holds important translational significance.

This study suggests that UTF1 likely acts as a regulator in maintaining functional stability between undifferentiated spermatogonia and Sertoli cells. Under physiological conditions, spermatogonial stem cells exhibit high UTF1 expression prior to differentiation, with subsequent downregulation upon differentiation initiation (Guo et al., 2018); this dynamic change is a hallmark of differentiation initiation. Conversely, under pathological conditions, aberrant UTF1 expression may lead to cellular retention in an undifferentiated state, as observed in cryptorchidism where elevated UTF1 is accompanied by decreased DAZL expression (Shakeel and Yoon, 2024). Moreover, aberrant expression of UTF1 and SOX9 in NOA may mediate disordered coupling between germ cells and Sertoli cells *via* POU5F1 (Roshan et al., 2024), consistent with the coordinated multi-cell-type changes observed upon *Utf1* deficiency in our study. Existing literature indicates that a core mechanism underlying NOA is the imbalance between spermatogonial stem/progenitor cell maintenance and the differentiation switch, which subsequently affects Sertoli cell function and the local signaling environment (Liu W. et al., 2024). Our study observed impaired early differentiation, accumulation of cells at later stages, and enhanced cell-cell communication following *Utf1* deficiency, suggesting that spermatogenic initiation is compromised while the differentiation program paradoxically advances and becomes arrested. This paradox may reflect a scenario in which, despite the failure of proper differentiation initiation, accumulated mitogenic and differentiative signals compel cells to enter downstream programs without the requisite molecular competence, ultimately leading to developmental arrest at later stages. Combined with evidence of aberrant Wnt signaling (Zeng et al., 2023; Wang et al., 2024), these findings indicate that UTF1 is involved not only in differentiation initiation but also in the orderly completion of the differentiation program.

ACTRT2 and AKAP4 stand out not only due to repeated algorithmic screening but more importantly because they align closely with different stages of spermatogenesis. Recent studies show that ACTRT2 is involved in spermatogonial iron homeostasis and mitochondrial integrity, and its loss increases susceptibility to ferroptosis in spermatogonia (Chen et al., 2025), consistent with our observation of its persistent downregulation in NOA-like states during early-to-mid pseudotime, suggesting a role in spermatogenesis initiation and early maintenance. In contrast, AKAP4 is a key structural protein of the sperm flagellar fibrous sheath; pathogenic variants can cause defects in sperm count, motility, and flagellar structure (Wei et al., 2023; Zhou et al., 2023) and have been used as protein markers for identifying rare sperm in NOA (Zhang et al., 2023), highlighting both mechanistic relevance and clinical potential. Although their functional contexts differ markedly—ACTRT2 operating at the level of metabolic homeostasis in early spermatogonia and AKAP4 functioning as a structural scaffold in mature sperm—their shared enriched pathways converge on microtubule nucleation, microtubule dynamics regulation, kinetochore–microtubule attachment, cytoskeletal remodeling, and flagellar assembly. This suggests that ACTRT2 and AKAP4 are not independent markers but collectively reflect a continuous pathological axis in NOA-from early spermatogonial dysregulation to late-stage sperm structural failure. Spermatogenesis, especially meiosis and spermiogenesis, critically depends on ordered microtubule organization and cytoskeletal remodeling: the former ensures accurate chromosome segregation and cell division, while the latter contributes to manchette formation, nuclear shaping, and flagellar assembly (Gao et al., 2025; Stathatos et al., 2025; Takeda et al., 2025). Thus, the ACTRT2-AKAP4 gene pair captures both early spermatogenic vulnerability and late sperm structural maturation failure, linking disease stratification, molecular markers, and cytological abnormalities, and may provide a basis for NOA risk assessment, residual spermatogenesis prediction, and patient stratification. Testosterone, mainly synthesized by Leydig cells, is essential for the conversion of round spermatids to elongated spermatids. In this study, serum testosterone levels were significantly lower in Utf1cKO mice than in controls at 35 dpp, suggesting that UTF1 deficiency may affect both spermatogenic and endocrine functions of the testis. This decrease may further impair spermatogonial proliferation and differentiation, potentially interacting with the spermatogenic defects observed in Utf1cKO mice. However, whether UTF1 directly regulates Leydig cell testosterone synthesis remains undetermined, and the reduced testosterone level may reflect abnormal testicular development, altered Leydig cell function, or a disturbed local microenvironment.

Our study identified that downregulation of ACTRT2 and AKAP4 is accompanied by immune microenvironment abnormalities and reprogramming of cell-cell communication. Existing studies have demonstrated that testicular immune homeostasis is essential for spermatogenesis, and its disruption can directly impair germ cell development (Li et al., 2024; Yang K. et al., 2025). In this study, increased infiltration of regulatory T cells (Tregs), myeloid-derived suppressor cells (MDSCs), and macrophages was observed, correlating negatively with ACTRT2 and AKAP4 expression, suggesting that NOA may be characterized by an inefficient immunosuppressive state (Sadek et al., 2024). Concurrently, communication axes such as Dll3-Notch, Grn-Sort1, and App-Cd74 were enhanced in the *Utf1* ^cKO^ group. Among these, Notch signaling is involved in cell adhesion and survival (Kamińska et al., 2024), while the APP-CD74 axis is associated with stress responses (Liu B. et al., 2024). Given that these signaling changes occur against a backdrop of severely compromised spermatogenic tubules and progressive germ cell loss, they likely represent compensatory or pathological rewiring within the damaged microenvironment rather than effective homeostatic maintenance, suggesting that while the initial impact of Utf1 deficiency originates from the spermatogenic program, the eventual phenotype is amplified through microenvironmental remodeling.

Overall, *Utf1* conditional knockout mice exhibited impaired testicular development and compromised spermatogenic function. On this basis, starting from the screening of key genes, we performed an integrative analysis of NOA-related spermatogenic abnormalities by combining developmental trajectories, microenvironmental changes, and structural processes. We found that ACTRT2 and AKAP4 are not only stably downregulated in NOA but also associated with cytoskeletal, immune, and differentiation abnormalities, with mutual support between human data and animal model results.

Nevertheless, this study has limitations, including a relatively small sample size, potential cross-species integration bias, and a lack of causal validation. In addition, although ACTRT2 and AKAP4 were validated by PCR, qRT-PCR and Western blot validation of UTF1 itself was not performed, because UTF1 served primarily as an upstream mechanistic entry point for model construction and candidate gene discovery rather than as a downstream marker to be validated. Future studies are still needed to confirm UTF1 expression changes at the mRNA and protein levels. The direct regulatory relationship between ACTRT2/AKAP4 and UTF1, as well as their functional links to aberrant communication axes, warrants further investigation. In particular, no rescue experiment was performed in this study; therefore, the association between UTF1 and ACTRT2/AKAP4 remains correlative rather than causally established. ACTRT2 and AKAP4 should be regarded as candidate downstream effector molecules associated with spermatogenic failure under UTF1 dysfunction, rather than confirmed direct transcriptional targets of UTF1. Future studies involving rescue experiments, promoter or enhancer reporter assays, and ChIP-seq or CUT&Tag analyses are required to directly define whether and how UTF1 regulates ACTRT2 and AKAP4. In addition, although the machine-learning models were constructed with cross-validation in the training cohort, their performance in the independent validation cohort was limited, with SVM-RFE showing a low AUC and the models tending to classify all validation samples as controls. This may be related to the small sample size and marked class imbalance of the validation cohort (16 NOA vs. 4 control samples), as well as cross-platform batch effects. Therefore, the predictive models should be considered exploratory, and their clinical utility requires further validation in larger and more balanced cohorts. Moreover, exploratory Spearman correlation analysis between gene expression and available clinical parameters in the training cohort, including serum FSH level, Johnsen score, and testicular volume, revealed only non-significant correlation trends for ACTRT2 and AKAP4 and no evident correlation for UTF1, which may be attributable to the limited number of samples with available clinical information (n = 19–20) and the heterogeneity of NOA; because corresponding clinical data were unavailable in the validation cohort, this analysis could not be independently verified. Future studies incorporating clinical samples and multi-omics approaches are needed to further evaluate the diagnostic and predictive value of these candidate markers and to elucidate the UTF1-mediated mechanisms regulating spermatogenesis. In the longer term, the development of non-invasive or minimally invasive diagnostic tools based on ACTRT2 and AKAP4 expression profiles may offer clinically actionable strategies for NOA risk stratification and sperm retrieval outcome prediction.

To elucidate the role of UTF1 in testicular development and spermatogenesis, this study established a germ cell–specific Utf1 conditional knockout (Utf1cKO) mouse model. The results demonstrated that Utf1 deficiency was closely associated with spermatogenic dysfunction and microenvironmental remodeling. Integrative analysis further identified ACTRT2 and AKAP4 as potential diagnostic biomarkers of this process. Collectively, these findings revealed a critical role of UTF1 in maintaining normal spermatogenesis and provided new insight into the molecular basis of NOA, supporting its potential value in disease stratification and targeted intervention. However, as these findings are primarily derived from experimental models and integrative analyses, their clinical applicability requires further validation in larger, well-characterized patient cohorts.

## Data Availability

The datasets presented in this study can be found in online repositories. The names of the repository/repositories and accession number(s) can be found below: https://www.ncbi.nlm.nih.gov/, GSE145467; https://www.ncbi.nlm.nih.gov/, GSE45887.

## Glossary

ACTRT2: actin-related protein T2
AKAP4: A-kinase anchoring protein 4
CASA: computer-assisted sperm analysis
Ctrl: control
DEGs: differentially expressed genes
DsigDB: Drug Signatures Database
dpc: days post coitum
dpp: days postpartum
GEO: Gene Expression Omnibus
GO: Gene Ontology
GSEA: Gene Set Enrichment Analysis
hdWGCNA: high-dimensional weighted gene co-expression network analysis
HE: hematoxylin and eosin
HL: Hosmer-Lemeshow
HVGs: highly variable genes
IHC: immunohistochemistry
KEGG: Kyoto Encyclopedia of Genes and Genomes
KMRGs: key module-related genes
MCODE: Molecular Complex Detection
MDSCs: myeloid-derived suppressor cells
miRNA: microRNA
MSigDB: Molecular Signatures Database
NK: natural killer
NOA: non-obstructive azoospermia
PCA: principal component analysis
PCNA: proliferating cell nuclear antigen
PPI: protein-protein interaction
RF: Random Forest
scRNA-seq: single-cell RNA sequencing
ssGSEA: single-sample Gene Set Enrichment Analysis
SVM-RFE: support vector machine-recursive feature elimination
TESE-ICSI: testicular sperm extraction combined with intracytoplasmic sperm injection
TF: transcription factor
Tregs: regulatory T cells
UMAP: uniform manifold approximation and projection
UMI: unique molecular identifier
UTF1: undifferentiated embryonic cell transcription factor 1
WT: wild-type
